# Wastewater leakage in West Texas revealed by satellite radar imagery and numerical modeling

**DOI:** 10.1038/s41598-019-51138-4

**Published:** 2019-10-10

**Authors:** Weiyu Zheng, Jin-Woo Kim, Syed Tabrez Ali, Zhong Lu

**Affiliations:** 1 Roy M. Huffington Department of Earth Sciences, Southern Methodist University, Dallas, Texas USA; 2 AIR-Worldwide, Boston, Massachusetts, USA

**Keywords:** Natural hazards, Hydrology

## Abstract

Wastewater, a byproduct of oil and gas production, is injected into disposal wells. Using Interferometric Synthetic Aperture Radar (InSAR) to observe ground deformation in the Ken Regan field, West Texas, we detected surface uplift that occurred near a wastewater disposal well from 2007 to 2011. High correlation between the observed deformation and the injection volume suggests that the uplift was caused by wastewater disposal in the well. Inverse elastic models were first used to calculate the injection depth and volume. Given the initial estimates of wastewater injection, forward poroelastic finite element models were applied to simulate stress/strain and displacement fields and to estimate the effective injection volume and depth, so as to ultimately understand the subsurface geomechanical processes and provide insight into the local hydrologic properties of the strata in the well location. Results from both elastic and poroelastic models indicate that the effective injection depth is much shallower than the depth reported to the Texas Railroad Commission (RRC). The most reasonable explanation is that the well was experiencing leakage due to casing failures and/or sealing problem(s). The Rustler Aquifer, within the zone of the effective injection depth, has been used as a source of freshwater for irrigation and livestock; wastewater leaked into this aquifer may possibly contaminate that freshwater. Our analysis that exploits remote sensing data and numerical models provides a clue as to understanding the subsurface hydrogeological process responding to the oil and gas activities and an indirect leakage monitoring method to supplement current infrequent leakage detection.

## Introduction

Wastewater, also referred to as “produced water” or “oilfield brine”, is a byproduct of oil and gas production. Oil and gas are pumped out with wastewater and then separated by going through a separation phase or by chemical treatment. The produced wastewater typically contains a large amount of sodium chloride as well as possibly toxic or radioactive chemicals depending on the properties of the producing rock formations^1^. Small quantities of residual hydrocarbons and industrial substances used in the well construction could also be included in the wastewater. Therefore, wastewater should be safely treated to avoid air, potable water and/or surface pollution. Predominantly, it is injected into underground porous zones which should be sealed above and below by unbroken, impermeable rock layers following the safety regulations of the state and federal agencies. The injection zones should be sufficiently deep (typical range is from 500 to 3,000 m in depth) in order to mitigate the contamination of shallow groundwater aquifers. However, approximately 5% of the oil-field related wastewater in the United States is discharged to the environment^2^, posing health risks, environmental contamination, and negative ecological impacts. There are many potential pathways for the wastewater to enter surface and groundwater, including spills from pipelines or tanker trucks transporting the wastewater, leakage and overflows from wastewater storage ponds, and upward migration of the fluids through the subsurface or through failed injection well casings^3^. Unlike the visible spills at the surface, subsurface leakages are usually harder to detect. Mechanical integrity tests that examine internal and external mechanical components of the well function are required every five years to ensure there is no significant leak in the well according to the regulations of U.S. Environmental Protection Agency (EPA). However, those infrequent tests could be, and sometimes are,augmented by alternative approaches such as *in-situ* fluid pressure measurements^4^ to monitor the underground processes to help detect the leakage as quickly as possible.

**Figure 1:**
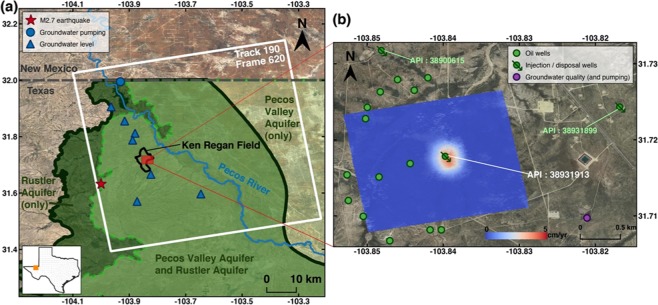
Study area. (**a**) Coverage of the ALOS PALSAR scenes used (white box). Black line shows the boundary of the Ken Regan field. Dark green line and light green line represent the boundaries of the Rustler Aquifer (subcrop) and Pecos Valley Aquifer in Texas, respectively. Red star represents the epicenter of the M2.7 earthquake that occurred in May 2018. Blue circle represents the groundwater well for livestock drawing from the Rustler Aquifer in this area. Blue triangles are groundwater wells which provide groundwater leveling records. (**b**) Vertical deformation rate (cm/yr) (in a red box of **a**) estimated from InSAR. Green circles with and without arrows indicate active injection/disposal wells in the Ken Regan field and oil production wells within 1.5 km from the deformation center during the research period, respectively. Purple circle represents the groundwater well (state well no. 4618201) which provides groundwater quality records. Dataset: © JAXA/METI ALOS PALSAR L1.0 2007–2011. Accessed through ASF DAAC 18 March 2018. The background images of (**a**,**b**) were from Landsat obtained from the EarthExplorer https://earthexplorer.usgs.gov provided by the United States Geological Survey (USGS). The figures were created using open-source software QGIS 3.6^40^.

The Ken Regan field, located in northern Reeves County, West Texas and within the Delaware Basin, produces hydrocarbons from the Delaware (Olds) sandstone of the upper Bell Canyon Formation^5^, which overlies the Cherry Canyon and Brushy Canyon Formations successively. These three formations, deposited in Guadalupian time of the Permian Period, comprise the Delaware Mountain Group, which contains more than 260 hydrocarbon reservoirs and has produced a large amount of oil and gas^6,7^. Then in Ochoan time, the sandstone and shale of the Delaware Group were covered by evaporites and limestone of the Castile Formation, which were in turn covered by evaporites interbedded with limestone, dolomite, sand, and shale of the Salado and Rustler Formations, which sealed and preserved the hydrocarbons. Partly dissolved dolomite, limestone, and gypsum of the Rustler Formation host the Rustler Aquifer^8^ (Fig. 1). All deposition occurred in a marine environment until the Jurassic Period, after which the area was uplifted above sea level and underwent erosion and subaerial deposition, creating the Delaware Basin. In Quaternary time, the climate became more arid, and deposition of silts, sands, and gravels from surrounding high areas formed Cenozoic Alluvium^9^, in which the water-bearing sediments host the major unconfined aquifer in West Texas: the Pecos Valley Aquifer (Fig. 1a). The stratigraphy of the geologic settings is shown in Table 1.

**Table 1:** Stratigraphy of the study area.

Geologic layers	Depth (m)	Layers in three-layer model	Hydraulic conductivity (six-layer model)	Hydraulic conductivity (three-layer model)
Cenozoic Alluvium	0–50	Caprock	3 × 10^−5^ m/s	5 × 10^−16^ m/s
Rustler Formation	50–200		3 × 10^−6^ m/s	
Salado Formation	200–500		1 × 10^−14^ m/s	
Castile Formation	500–1020		5 × 10^−16^ m/s	
Bell Canyon	1020–1350	Injection Zone (Reported injection point: 1040 m)	1 × 10^−10^ m/s	1 × 10^−10^ m/s
Cherry Canyon	1350–1650			
Brushy Canyon	1650–2200	Base rock	5 × 10^−13^ m/s	5 × 10^−13^ m/s

The injection/disposal well American Petroleum Institute (API) No. 38931913 is located in the Ken Regan field (31.718°N 103.84°W). Originally completed for oil and gas production in 1989, by 1992, the well was granted a permit to dispose previously oil and gas produced wastewater by the Texas Railroad Commission (RRC), Texas’ primary oil/gas-regulatory agency. In 2001, oil and gas production ceased and the well became a dedicated wastewater disposal well. Total oil and gas production from this well are more than 8,000 barrels and 100,000 thousand cubic feet (MCF), respectively. As a disposal well, it played an important role in the Ken Regan field, taking in 44% of the total volume injected into the whole field from 2007 to 2011. After 2015, the injection rate decreased greatly, accommodating only 0.6% of the total volume injected in the field. In 2017, the injection operations at the well were concluded. According to the H-10 form provided to the Texas RRC, the injection depth is reported to be 1,040 m, where the Bell Canyon Formation lies (Table 1).

Generally, as the pore pressure builds up inside a deep wastewater injection zone, the pressure increases can propagate to other surrounding underground and overlying rock/soil layers, resulting in surface uplift^9^. When basement faulting exists, the decrease of the effective normal stress on the adjacent faults can also increase the chances of failure and cause induced seismicity^10^. However, there have been only a few ways to monitor the spatial pattern of the surface displacement caused by oil and gas activities in remote areas. *In-situ* methods to measure surface uplift in the well vicinity are labor-intensive, time-consuming, and sparsely distributed. Moreover, in many cases, it is challenging to pinpoint hydrocarbon production or wastewater injection wells that have experienced such surface displacement and are thus candidates for increased attention to ensure safe operation.

Interferometric Synthetic Aperture Radar (InSAR) is an effective tool for mapping ground deformation with centimeter to millimeter level precision and meter level resolution^11^. InSAR has been successfully used for monitoring surface deformation induced by wastewater injection and other oil field related fluid injection processes, and has proven its capacity to measure small to large induced deformation over localized to regional spatial scales^12–14^. Both inverse elastic and forward poroelastic models have been constructed to simulate surface deformation induced by wastewater injection. Although elastic models may not be fully realistic and cannot be applied to all the geological settings, they can still provide insight into the subsurface geomechanical process^15^. Poroelastic models are believed to more closely approximate reality and have performed well in many known cases^12^. However, it is difficult to get precise hydrogeomechanical parameters of various geologic materials in the poroelastic models without obtaining samples from the subsurface or complete well logs. Poroelastic models are usually used to simulate the properties of the strata^12,16^ but seldom used for analyzing unexpected underground processes such as wastewater leakage and subsurface fluid migration.

In this paper, we used data acquired by the Advanced Land Observation Satellite (ALOS) Phased Array type L-band Synthetic Aperture Radar (PALSAR) from 2007 to 2011 to generate InSAR images and analyze the time series deformation induced by wastewater disposal at the API No. 38931913 well. Elastic Mogi^17^ and Okada^18^ models were utilized to provide the initial estimates of geomechanical processes that were further analyzed using three-dimensional, finite element based, poroelastic models via Defmod^19^. Initially, six-layer models (Cenozoic Alluvium - Rustler Formation - Salado Formation - Castile Formation - injection zone - base rock) were employed to test and refine the local hydrologic properties. With the displacement-driven refinement, we next used a three-layer model (caprock - injection zone - base rock) to investigate the underlying geomechanical processes, which could provide information about undesired subsurface processes such as wastewater leakage and fluid migration.

## Results

### InSAR time series analysis

**Figure 2:**
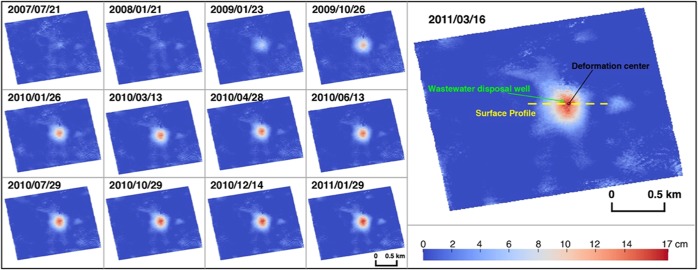
Time series cumulative vertical deformation maps from 2007/01/18 to 2011/03/16 over the study area. The reference image for 2007/01/18 is omitted. Green circle represents the API No. 38931913 wastewater disposal well. Black circle represents the deformation center. Yellow dash line shows the surface profile of the total vertical deformation plotted in Fig. 5(d).

InSAR processing was used to generate time series deformation maps from January 18, 2007 to March 16, 2011 (Fig. 2 and Methods section. 5). We have detected an area of persistent uplift (Fig. 2). The uplift occupies area with a radius of 250 m, which is relatively localized compared with other km-sized fluid injection induced deformation^12–15^. The cumulative vertical deformation reaches nearly 17 cm during 2007–2011. However, the study area has been seismically quiet according to USGS and TexNet earthquake catalogs; the epicenter of the nearest earthquake, a M2.7 event that occurred in 2018, is more than 10 km away from this area (red star in Fig. 1a). The comparatively long distance suggests the seismicity is irrelevant with the observed small-radius uplift. Only one wastewater disposal well (API No. 38931913) is located within the uplift area, and other injection/disposal wells active during the research period are distributed about 2 km away from the deformation center (Fig. 1b). There are active oil production wells within a distance of 1.5 km (Fig. 1b), but the total production volume of all 14 wells is less than 1% of the injection volume in the wastewater disposal well. Thus, we focus our attention on the correlation between the uplift and the wastewater injection at API No. 38931913.

The deformation center lies ~70 m southeast of the disposal well (Fig. 2). Most vertical wells are not truly vertical, but are in fact tilted, allowing the pressurized wastewater injection to flow laterally some distance from the surface wellbore location. The southeastward offset between the deformation center and the disposal well also implies the direction of the groundwater flow. Due to the lack of groundwater stations in the study area, it is difficult to determine the direction of local groundwater flow within different layers of aquifer systems. Sharp^20^ suggested a probable southeastward direction of the nearby regional flow system (Salt Basin - Toyah Basin - Pecos River system), consistent with our observation. Groundwater level measurements at wells in the Pecos Valley Aquifer (Fig. 1a) provided by the Texas Water Development Board (TWDB) also indicate a southward direction along with an eastward component. However, the local flow direction within the Rustler Aquifer in the Delaware Basin can be affected by variations in the potentiometric surface resulting from oil-related production activities (e.g., water production from hydrocarbon activities) as well as local features produced by evaporite dissolution and collapse.

**Figure 3:**
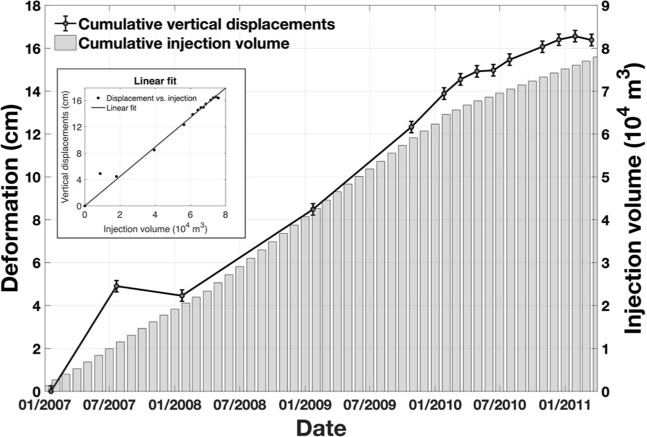
Comparison of the cumulative wastewater injection volume (gray bars) and time-series cumulative vertical deformation at the deformation center (black line). The error bars show uncertainties obtained in the time-series analysis. Inset: Linear fitting of the cumulative vertical deformation and the cumulative wastewater injection volume, assuming the displacement is zero when the injection starts.

The cumulative peak vertical displacement is highly correlated with the cumulative injection volume from January 2007 to early 2011 (Fig. 3). Both the displacement rate and the injection volume rate decreased after March 2010, suggesting the displacement responded almost instantaneously (within the ALOS repeat cycle of 46 days and the 1-month interval of the RRC injection data) to the wastewater injection. Assuming the displacement is zero when the injection starts, the cumulative vertical displacement and cumulative injection volume show high correlation in the linear fitting (inset of Fig. 3). The ratio between injection volume (10^4^ m^3^) and vertical displacement (cm) is 2.24 with an R-squared value of 97.5%. The high correlation indicates the ground surface was heaving due to the wastewater injection in the No. 38931913 disposal well. The Sentinel-1A/B images acquired over this region have not been able to detect any deformation during 2015 to 2019 when the injection decreased the rate and finally ceased, further supporting the conclusion that the ground uplift was caused by wastewater injection.

### Inverse elastic models

**Figure 4:**
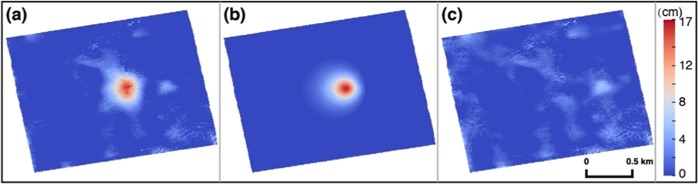
Mogi model of cumulative vertical deformation from 2007/01/18 to 2011/03/16. (**a**) Observation, (**b**) simulated deformation, and (**c**) residual.

Due to the instantaneous and linear response between the fluid injection and displacement, the elastic models are expected to be reasonable for estimating the parameters related to geomechanical processes in the injection zone and surrounding strata despite some limitations^15^. As inverse Mogi models are easy to implement, they were constructed to simulate the time series deformation observed by InSAR and estimate the effective injection depth and volume. The Mogi model to calculate an analytical solution for surface deformation due to a point source in an elastic half space performs well (Fig. 4) with a root mean square error (RMSE) of 1.46 cm, but the total effective injection volume derived from the Mogi model is only 27% of the reported injection volume. And notably, the modeled effective average injection depth was only 177 m, which is much shallower than the reported injection depth of 1,040 m. Okada models were also utilized to model the deformation and check the parameters from the Mogi model. The total effective injection volume at the rectangular dislocation source (217 × 343 m) was just 21% of the reported injection volume. The effective injection depth derived from the Okada model averages 186 m, again much shallower than the reported injection depth.

### Forward poroelastic models

**Figure 5:**
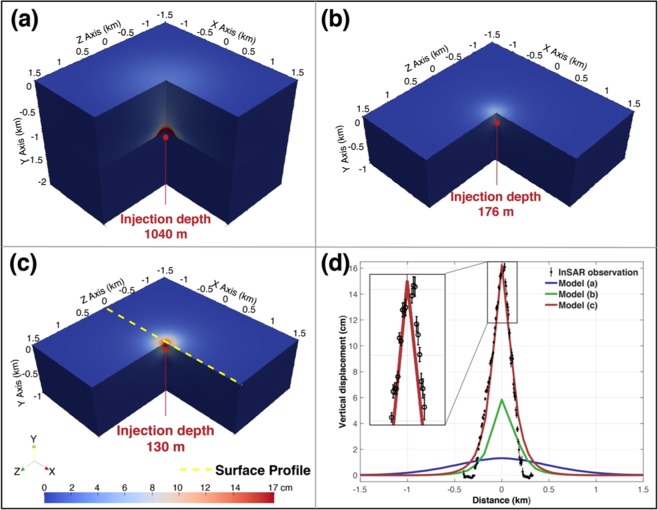
Final displacement fields and surface profiles of the forward poroelastic models. Orientation axes show the optic angle of the deformation field. Red dots represent the injection depths. Yellow dashed line shows the surface profile plotted in (**d**). (**a**) Three-layer model using reported injection depth and volume. (**b**) One-layer (caprock) model using Mogi-derived depth and volume. (**c**) Best-fit model derived by poroelastic modeling. (**d**) Comparison of vertical displacement surface profiles of InSAR observation and the poroelastic models. The error bars of the InSAR observation show uncertainties obtained in the time-series analysis. The upper part of the comparison is enlarged in the inset.

Finite element models were applied to model the total deformation map (2007/01/18 – 2011/03/16) by taking into account the poroelastic effects in the pressure and displacement fields. With the refinement of the six-layer models (Cenozoic Alluvium - Rustler Formation - Salado Formation - Castile Formation - injection zone - base rock) based on observed displacement (see Discussion 3.1), a three-layer (caprock - injection zone - base rock) poroelastic model with reported injection volume and reported depth was utilized to simulate the total surface deformation (Fig. 5a). The profile of the surface vertical displacement in comparison with the profile of the observation is shown in Fig. 5(d). The deformation simulated based on the reported injection information (injection depth: 1,040 m) is broader and its magnitude is smaller (blue line in Fig. 5d) than our InSAR observation (black circles in Fig. 5d), indicating that the underlying geo-mechanical process cannot be simply interpreted by the reported injection depth and volume. The huge difference between the simulation and the observation made it difficult to find the best solution of effective volume and depth when using the reported injection information as the first step. To avoid time-consuming computation in the iterative scheme of finite element models, we needed better initial estimates of the solution, which could be provided by the elastic models. We thus simulated the poroelastic deformation using source parameter from the Mogi (injection depth: 176 m). As the Mogi-derived depth is within the caprock, we modeled a one-layer (caprock only) poroelastic scenario for simplicity (Fig. 5b). The spatial size of the poroelastic-modeled uplift is similar to the Mogi-derived deformation (green line in Fig. 5d) but its magnitude is smaller, which is consistent with the comparison of elastic and poroelastic models by Samsonov^15^. In order to further refine this solution and obtain a best-fit model, we tried a range of effective injection depth and volume in both the three-layer and one-layer models. The best-fit parameters are found in the one-layer model with an effective injection volume of 4.4 × 10^4^ m^3^ (57% of the reported injection volume) and an effective injection depth of 130 m, even shallower than the depth derived from the inverse elastic models (Fig. 5c,d).

The shallow effective injection depth derived from both inverse elastic models and forward poroelastic models suggests that part of the injected wastewater somehow leaked into a shallower aquifer in the caprock and induced localized surface uplift, while the remaining wastewater may have diffused away in the injection zone or other strata.

## Discussion

### Insights into local hydrologic properties

The hydraulic conductivities of the Rustler Formation and Cenozoic Alluvium are generally high (>10^−6^ m/s). We have analyzed multiple six-layer (Cenozoic Alluvium - Rustler Formation - Salado Formation - Castile Formation - injection zone - base rock) poroelastic models with varying injection depths and volumes, but none generated an area of uplift as localized as the observation, and the magnitude of uplift failed to reach even 10 cm (versus the ~17 cm observed). There are five hydrologic units in the Rustler Formation with 11 hydro-stratigraphic divisions^21^. Theoretically, we can refine the model with those divisions to seek solutions for the uplift. However, due to the lack of information in the well log, the local stratigraphy cannot be described with an accuracy of 10-meter level. The failure in generating localized deformation are reasonable because high hydraulic conductivities render rapidly spreading fluid (or pore pressure). The pressure change cannot be accumulated, thus no obvious surface deformation can be induced. A confined aquifer somehow existing within the Rustler Formation and/or Cenozoic Alluvium could help confine the wastewater, but it is difficult to explain how the fluid flows into, but not out of, the aquifer. Besides, the confined aquifer would behave similarly to locally impermeable material in terms of induced deformation. Therefore, we made the refinement of the local hydrologic properties assuming that the Rustler Formation and Cenozoic Alluvium are locally impermeable and perform as caprock.

We assumed the uplift area is a circle for the simulation, and thus the hydrologic properties of the strata can be considered isotropic. However, the trend of the surface profile in the east side of the deformation center is slightly larger than the west side, which indicates the local lateral properties could be slightly anisotropic. Anisotropic lateral properties would be consistent with the observed center of the deformation offset from the wastewater disposal well.

### Poroelastic models based on the reported injection depth

**Figure 6:**
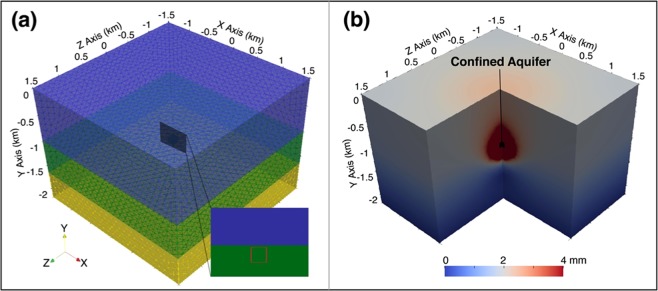
Three-layer poroelastic model with a confined aquifer. (**a**) Geometry and mesh of the finite element model. Blue, green and yellow brick represent the caprock, injection zone, and the base rock, respectively. Enlarged rectangle shows the location of the surrounding impermeable material (red square), inside of which is a confined aquifer. Orientation axes show the optic angle of the deformation field. (**b**) The final displacement field of (**a**) using reported depth and volume. The black shape shows the location of the confined aquifer.

When constructing wells, low permeable substances, such as drilling mud that is used to aid the drilling of boreholes, could also be injected to depth. A possible mechanism for a localized surface uplift with deep injection is that a confined aquifer in the injection zone was formed by a surrounding layer of impermeable material and the injection coincidently occurs in the confined aquifer which prevents the wastewater from diffusing away. To evaluate this theory, we added a 10 m wide impermeable material into the injection zone in the three-layer poroelastic model to simulate a confined aquifer (Fig. 6a). The output of reported injection depth and volume in Fig. 6b shows a broader and significantly smaller uplift. Different shapes of the confined aquifer were simulated but none of them performs as well as expected. The non-localized deformation in these cases further proves that the effective injection depth should be shallower than the reported depth.

### Possible causes of the shallow effective injection depth

**Figure 7:**
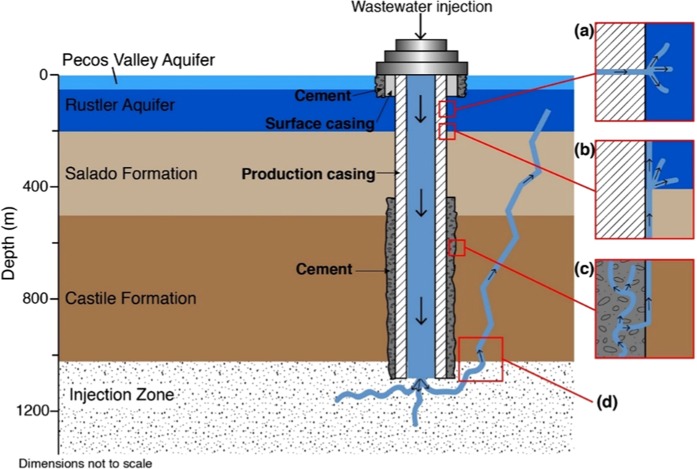
Stratigraphy, well completion, and possible pathways for upward migration of wastewater. Possible leakage caused by (**a**) failed production casing, (**b**) sealing problem - through the space between the casing and the wellbore, (**c**) sealing problem - through vertical channel in the faulty cement, and (**d**) fault and fracture systems. The figure was created using Adobe Illustrator CC (2015.0.0 release) licensed by Southern Methodist University.

According to the results of the best-fit poroelastic model, 57% of the reported injection volume leaks to the effective injection depth of 130 m (in the range of the Rustler Aquifer), inducing the localized surface uplift. The remaining 43% of the wastewater could have diffused away into the reported injection zone or other strata, causing negligible far-field deformation. Possible causes of the leakage (Fig. 7) could include: failure in the production casing, sealing problem, and fluid migration through the subsurface fractures^22^.

#### Failed production casing

The surface casing and the surrounding cement, built for protecting the underground source of drinking water - the Pecos Valley Aquifer (Fig. 7), is reported to extend to 77 m deep according to the casing records of the API No. 38931913 wastewater disposal well. The production casing along with surface casing and sealing cement in the 0–77 m deep are less likely to fail, but at the effective injection depth of 130 m, the production casing (typically made from carbon steel) and the inside tubing (ideally corrosion-resistant material) are subject to a corrosion accelerated by chemicals (e.g., Hydrogen Sulfide) present in both the Rustler Formation and the injected wastewater. In 2007, the well was about 20 years old. Even a well originally constructed within safety parameters from materials that passed mechanical integrity tests could possibly experience later leakage due to a casing failure including corrosion, axial cracking, and joint/coupling problems.

A direct conjecture is that the production casing failed at the effective injection depth of 130 m allowing 57% of the wastewater to flow out at this failed section (Fig. 7a). The failed section does not need to be a complete mechanical failure, which would allow all the wastewater to leak at the effective injection depth. Instead, small cracks (or holes in corroded pipes) are sufficient, which is the more likely scenario. With high pressure during the injection and small perforations in the production casing, the remaining 43% of the wastewater could flow down through the wellbore and diffuse away into the reported injection zone. In this case, the relationship between displacement rate and cumulative injection volume rate may not be linear but more complicated, with (at least) both the pressure and the area of failed wellbore section considered as important factors.

#### Sealing problems

Sealing problems may be caused by voids between production casing/cement and surrounding sediments (Fig. 7b). In this case, the wastewater flows up from the reported injection zone along the wellbore either until the deepest permeable aquifer, into which the wastewater will diffuse, or until the void disappears. Considering the deepest permeable aquifer assumption, if we ignore the hypothesis for the 3-layer model that the Rustler Formation is impermeable, the wastewater will accumulate in the Rustler Formation as it is the first permeable aquifer (Fig. 7b). Nevertheless, the lowest layer inside the Rustler Formation is a permeable aquifer, not a confining bed, indicating the effective depth would be 200 m but not 130 m. It is coincident that the effective injection depth from the Okada model is ~200 m but that is meaningless because if so, the wastewater will diffuse rapidly at that aquifer and not induce a localized surface uplift. Thus, the upward migration of wastewater is less possibly to end at the deepest permeable aquifer, and the sealing problem can explain the shallow effective injection depth only if the voids between production casing/cement and surrounding sediments appear from 130 m depth to deeper.

The bottom part of the production casing is sealed by cement (Fig. 7). If the seal worsens over time or the well has not been sealed properly, after the wastewater is disposed into the injection zone, it may flow up along the vertical channel in the faulty cement (Fig. 7c). The flowing-up wastewater could either gather in the top of the cement or flow out into any part of the cement and then flow up along the voids between the cement and the surrounding sediments, where possible. It will not diffuse in the Salado and/or the Castile Formations, as they are not permeable aquifers, comparatively. However, the top of the cement to protect the bottom casing is reported to be 445 m, deeper than the effective depth, indicating a sealing problem in the cement cannot solely explain the leakage, but could perhaps provide an additional pathway for upward fluid migration in the bottom part of the well.

Leakage due to sealing problems can only be explained by the hypothesis that voids between production casing and surrounding sediments occur from 130–445 m combined with faulty cement and/or voids between cement and surrounding sediments present from 445 m to the reported injection zone, providing continuous pathways for wastewater to flow up from the reported depth to the effective injection depth.

#### Subsurface fractures

**Figure 8:**
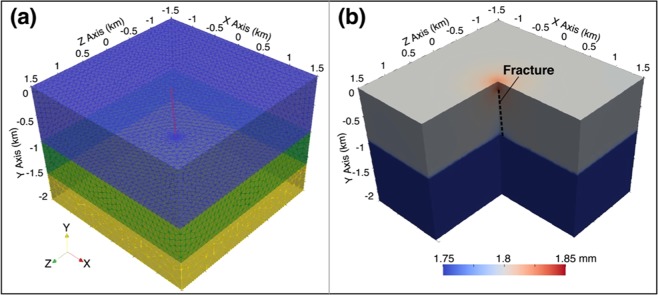
Three-layer poroelastic model with a fracture. (**a**) Geometry and mesh of the finite element model. Blue, green and yellow brick represent the caprock, injection zone, and the base rock, respectively. Red inclined cylinder (10 m radius) and the sphere (20 m radius) at the top of the cylinder represent the highly permeable pathway (the fracture) and the confined aquifer, respectively. Orientation axes show the optic angle of the deformation field. (**b**) The final displacement field of (**a**) using reported depth and volume. Black dashed line shows the location of the fracture, inclined to model the 70 m lateral distance from the surface wellbore to the surface deformation center.

Plenty of fault and fracture systems are located underlying the Delaware Basin, and have been thought to bring hydrologic communication between different layers of aquifer systems^23^. These fractures may provide pathways for upward migration of injected wastewater (Fig. 7d). To link the reported injection zone and the effective injection depth, we added a tube of highly permeable material with a radius of 10 m as a path to simulate a fracture into the three-layer poroelastic model (Fig. 8a). The inclination of the tube is positioned to model the 70 m distance between the deformation center and the wastewater disposal well. We also assumed that there is a high-permeability confined aquifer (a sphere with radius 20 m) at the effective injection depth to increase the attraction for the wastewater. However, the output displacement field in Fig. 8b indicates that the 20 m wide path still cannot draw as much wastewater as we expected (or the pore pressure change cannot accumulate to what we expected) if no other forces are added. This is despite the fact that fractures in reality should be much narrower than 20 m. Thus, leakage due to subsurface fractures cannot be the main cause of the localized uplift.

Other limitations of this explanation include the following. (1) If the injection completes, previously accumulated wastewater in the upward confined aquifer will spread back down into the injection zone via the same fracture(s) as the hydraulic conductivities of the fracture and injection zone are higher than the surrounding caprock. If so, we should see subsidence over time, which has not been observed from the Sentinel-1 A/B images acquired over the same area from 2015–2019. (2) Generally, actual fractures are distributed irregularly and randomly, which cannot induce the very circular surface uplift as observed.

Possible reasons for the leakage are therefore concluded to be failed production casing, sealing problem(s), or the combination effect of these two, while the leakage along subsurface fractures and resulting uplift are less plausible.

### Impacts of the leakage

The effective injection depth of 130 m is inside the Rustler Aquifer. The Rustler Aquifer is only used for irrigation and livestock and not municipal and domestic supply, due to the high concentrations of dissolved solids^24^. Although the drinking water may not be directly impacted, possible leakage into the Rustler Aquifer can pose potential risks to crops and livestock. There is only one single groundwater well pumping for livestock in this area from the Rustler Aquifer (blue circle in Fig. 1a). However, some groundwater from the Rustler Formation does eventually discharge into the Pecos River^25^. Upward leakage into the overlying strata could also happen^26^, impacting water quality in the overlying aquifers. Besides, the fault and fracture systems provide pathways for rapid water migration. Due to the void of groundwater wells into the Rustler Aquifer in the vicinity, we cannot check the nearby water quality in the Rustler Formation. However, the groundwater quality records at state water well no. 4618201 (31.71°N 103.821°W; purple circle in Fig. 1b) show an increase of dissolved sodium in the Pecos Valley Aquifer. The dissolved sodium increased 13 mg/L during 2007–2011 and 5 mg/L during 2011–2018, which could be related to wastewater injection (comparison of the water quality and the injection volume is provided in the supplementary information). As this well is used for livestock and the pumping has not been concluded, this reduced level of groundwater quality is still within acceptable parameters. The possible leakage of toxic fluids can bring health risks, environmental contamination, and negative ecological effects. As we have not observed clear subsidence since the injection operations concluded with Sentinel-1 results, the wastewater seems to disperse slowly, reducing the risk to some extent.

## Conclusion

In our study, InSAR has shown the capability to measure a localized surface displacement related to subsurface fluid injection. The surface uplift near the wastewater disposal well (API NO. 38931913) in the Ken Regan field is caused by wastewater disposal in this well. The inverse elastic Mogi model performs well to roughly estimate the effective injection depth and volume from the measured InSAR deformation. Defmod is effective in investigating the poroelastic subsurface processes. The combination of InSAR results and poroelastic models generated by Defmod gives a clue about hydrologic properties of the strata. The modeled effective injection depth (130 m) of this well is much shallower than the reported injection depth (1,040 m). A reasonable explanation is that the well has experienced leakage due to a failed production casing and/or sealing problem(s). Leakage into the Rustler Aquifer poses some risk, but maybe not be serious when the wastewater disperses slowly away as is believed to be the case. Our analysis that exploits InSAR observation and numerical models provides an indirect leakage monitoring technique to supplement current infrequent leakage detection methods.

## Methods

### InSAR processing

ALOS PALSAR data are used to detect ground deformation in the Ken Regan field. The area is covered with a sparse, short vegetation and is thus more likely to be coherent using L-band data. 14 images (ascending track: 190, frame: 620) from January 18, 2007 to March 16, 2011 were acquired to generate interferograms. We have applied a 1 × 2 m multilook window to maintain high resolution and coherence. Adaptive spatio-temporal filtering has been used to suppress noise components related to atmospheric artifacts. Because we have SAR datasets from an ascending track only, we cannot retrieve both the horizontal (east-west) and the vertical deformation. However, the observed the line-of-sight (LOS) deformation is dominated by the vertical deformation in the wastewater disposal well of the Delaware Basin^13^. We therefore convert LOS to the vertical deformation (more description in the Supplementary). We remove the topographic effects using 1-arcsec digital elevation model (DEM) data from the shuttle radar topography mission (SRTM)^27^. After removing topographic effects, 31 interferograms with high coherence (>0.4) were chosen for the time-series analysis using the Small Baseline Subset (SBAS) technique^28^. By minimizing the temporal and spatial baseline between the acquisitions required for applying the SBAS method, decorrelation artifacts can be further mitigated. The abundant multi-temporal InSAR observations over a small area (2 × 1.7 km) help separate signatures of deformation and atmospheric effects with the aid of spatio-temporal filtering.

### Inverse elastic models

We used Mogi modeling^17^ to simulate the surface deformation maps and estimate the corresponding injection volume and depth. This technique models the deformation from a point source in an elastic half-space, which is widely applicable in geophysical studies^29^ and has been used for modeling deformation caused by fluid injection^15^. Displacement induced by wastewater injection can be calculated according to (1):

1 $$\begin{array}{rcl}(\begin{array}{c}{u}_{x}\\ {u}_{y}\\ {u}_{z}\end{array}) & = & \Delta V\frac{(1-\nu )}{\pi {R}^{3}}(\begin{array}{c}x\\ y\\ d\end{array})\\ {\rm{R}} & = & \sqrt{{x}^{2}+{y}^{2}+{d}^{2}}\end{array}$$

where *x* and *y* are the distance in the *x* and *y* directions from the point to the injection well, *d* is the injection depth, *u*_*x*_, *u*_*y*_, *u*_*z*_ are the displacements in the *x*, *y*, and *z* directions, Δ*V* is the injection volume, *v* is the Poisson’s ratio, and *R* is the radial distance (distance between the source and the point whose coordinates are *x* and *y* at the surface). We determined the best-fit models and parameters by searching over the range of the parameters and minimizing the root mean square of the residuals.

In addition to the Mogi modeling, a horizontal Okada model^18^ with uniform opening in an elastic half-space was also applied to estimate injection depth. Okada models are usually used as the source model for earthquakes^30^ and volcanoes and have also been used for wastewater injection analysis^13^.

### Forward poroelastic model

Defmod^19^, an open source finite element code for modeling crustal deformation, was benchmarked and validated by Meng^31^ and has been successfully used to model earthquakes induced by fluid withdrawal and/or injection^31^ and to investigate deformation in a geothermal field^32^. In this study, we used the poroelastic module of Defmod to model the surface uplift due to wastewater injection. Trelis™ was used to generate the mesh required by Defmod. For each 3D model, more than 100,000 tetrahedral elements were generated in the mesh file; the surface area is 3 km × 3 km. The mesh file was plugged into Defmod^19^ to solve the coupled system of the momentum equation and the continuity equation in the discretized form:

2 $$\begin{array}{rcl}{K}_{e}u-Hp & = & f\\ {H}^{T}\dot{u}+S\dot{p}+{K}_{c}p & = & q\end{array}$$

where *K*_*e*_ and *K*_*c*_ are solid and fluid stiffness matrices; *H* is the coupling matrix; *S* is the compressibility matrix; *u* is the displacement field; *p* is the pressure field; *f* is the body force and *q* is the in/out flow^19^. The well log of the API No. 38931913 well only covers part of the Bell Canyon Formation, so properties (depth, Young’s modulus, Poisson’s ratio, hydraulic conductivity, etc.) of the underground layers can only be obtained from previous research^21,33–36^ and adjacent well logs^37–39^. There could be uncertainties of these properties as they are not directly acquired from on-site measurements; we would update the properties if more information from oil companies or field measurements can be gathered. As the Brushy Canyon is generally less permeable than the other two formations in the Delaware Mountain Group, we classified the Bell Canyon and Cherry Canyon both as injection zone and the Brushy Canyon as base rock. Although the Rustler Formation and Cenozoic Alluvium are comparatively permeable, we hypothesize that the hydraulic conductivities of these two formations in the vicinity are locally low because highly permeable materials render rapidly spreading fluid, making it nearly impossible to accumulate pressure change and induce surface deformation, while low hydraulic conductivities allow slowly dispersing fluid, cumulative pressure change and thus further localized surface deformation. This restriction can be derived from InSAR results and poroelastic models (discussed in 3.1). Therefore, we consider all formations above Bell Canyon as caprock for modeling purposes (Table 1).

## Supplementary information

Wastewater leakage in West Texas revealed by satellite radar imagery and numerical modeling

## Data Availability

The datasets generated during this study are available from the corresponding author upon reasonable request.
