# Supplementary material for: Wastewater leakage in West Texas revealed by satellite radar imagery and numerical modeling
Source: Sci Rep. 2019 Oct 10;9:14601. doi: 10.1038/s41598-019-51138-4 (PMC6787232; doi:10.1038/s41598-019-51138-4)
Supplement: Supplementary file 1 — Wastewater leakage in West Texas revealed by satellite radar imagery and numerical modeling [file 41598_2019_51138_MOESM1_ESM.pdf]

Wastewater leakage in West Texas revealed by satellite radar imagery and numerical modeling

Weiyu Zheng[1], Jin-Woo Kim[1], Syed Tabrez Ali[2], Zhong Lu[1*]

[1]Roy M. Huffington Dept. of Earth Sciences, Southern Methodist University, Dallas, Texas, USA

[2]AIR-Worldwide, Boston, Massachusetts, USA

* Corresponding author: zhonglu@smu.edu

Supplementary

Groundwater quality

Due to the lack of groundwater wells into the Rustler Aquifer in the vicinity, we were unable to check the water quality at the possible leakage point. However, the groundwater quality in the Pecos Valley Aquifer can reflect conditions in the Rustler Aquifer to some extent as a result of upward water migration. We compared the groundwater quality records from the nearest groundwater well into the Pecos Valley Aquifer (state well no. 4618201; 31.71˚ N 103.821˚ W) with the cumulative wastewater injection volume (Fig. S1). The injection rate was high from 2007 to 2011; the corresponding growth rate of dissolved sodium was also comparatively large (3.25 mg/(L·yr)). When the injection rate decreased after 2011, the growth rate of dissolved sodium also decreased (0.71 mg/(L·yr)). The similar trend of the dissolved sodium in the Pecos

Aquifer and the cumulative injection volume suggests that the wastewater injection may influence the groundwater quality in the Pecos Valley Aquifer.

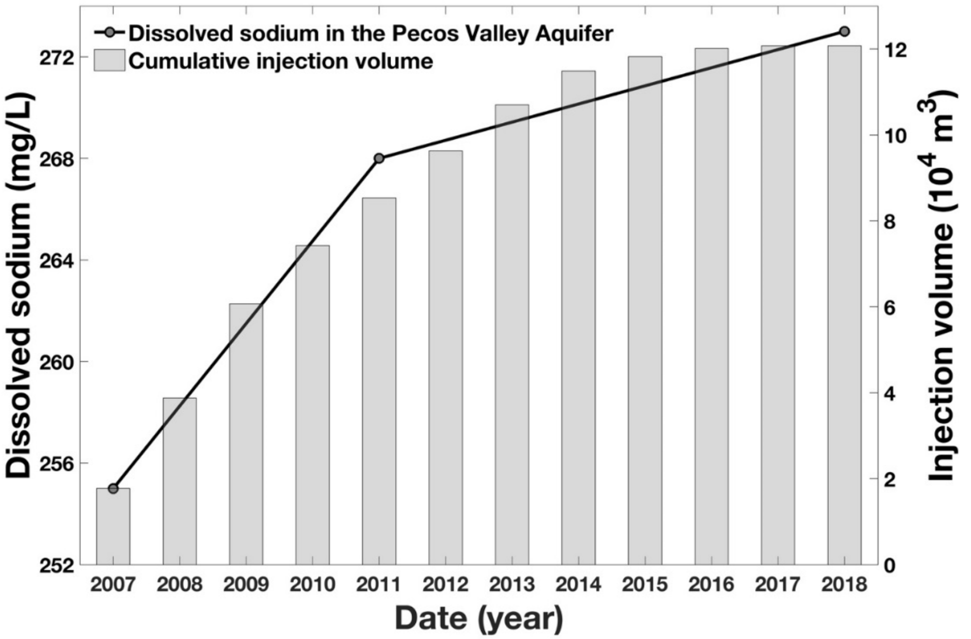

Figure S1. Comparison of the cumulative wastewater injection volume (gray bars) and the dissolved sodium in the Pecos Valley Aquifer (black line).

Conversion from LOS to vertical displacement

The vertical displacement d_{ver} can be calculated as:

$$d_{ver} = d_{LOS}/\cos\theta \quad \text{(S1)}$$

where d_{LOS} is the LOS displacement and θ is the incidence angle. The lack of descending track data makes the horizontal displacement unavailable, causing uncertainties in above conversion. It has been shown that the horizontal deformation is less than ~20% of the vertical deformation in wastewater injection cases in the region[13].
